# Osteoimmunology: The Crosstalk between T Cells, B Cells, and Osteoclasts in Rheumatoid Arthritis

**DOI:** 10.3390/ijms25052688

**Published:** 2024-02-26

**Authors:** Mei Yang, Lei Zhu

**Affiliations:** 1 Department of Pharmacology, Institute of Basic Medical Sciences, Chinese Academy of Medical Sciences and School of Basic Medicine, Peking Union Medical College, Beijing 100005, China; ym258025ym@163.com; 2 Medical Epigenetics Research Center, Chinese Academy of Medical Sciences, Beijing 100005, China

**Keywords:** osteoimmunology, rheumatoid arthritis, osteoclasts, T cell, B cell

## Abstract

Rheumatoid arthritis (RA) is an ongoing inflammatory condition that affects the joints and can lead to severe damage to cartilage and bones, resulting in significant disability. This condition occurs when the immune system becomes overactive, causing osteoclasts, cells responsible for breaking down bone, to become more active than necessary, leading to bone breakdown. RA disrupts the equilibrium between osteoclasts and osteoblasts, resulting in serious complications such as localized bone erosion, weakened bones surrounding the joints, and even widespread osteoporosis. Antibodies against the receptor activator of nuclear factor-κB ligand (RANKL), a crucial stimulator of osteoclast differentiation, have shown great effectiveness both in laboratory settings and actual patient cases. Researchers are increasingly focusing on osteoclasts as significant contributors to bone erosion in RA. Given that RA involves an overactive immune system, T cells and B cells play a pivotal role by intensifying the immune response. The imbalance between Th17 cells and Treg cells, premature aging of T cells, and excessive production of antibodies by B cells not only exacerbate inflammation but also accelerate bone destruction. Understanding the connection between the immune system and osteoclasts is crucial for comprehending the impact of RA on bone health. By delving into the immune mechanisms that lead to joint damage, exploring the interactions between the immune system and osteoclasts, and investigating new biomarkers for RA, we can significantly improve early diagnosis, treatment, and prognosis of this condition.

## 1. Introduction

Earlier this century, the term “Osteoimmunology” was coined by researcher Aaron to describe the intricate relationship between the skeletal system and the immune system, establishing a connection between abnormal bone metabolism and immune system disorders [1]. Osteoimmunology encompasses various diseases, including rheumatoid arthritis (RA), osteoporosis, ankylosing spondylitis, periodontitis, and bone infections. RA, a significant autoimmune inflammatory disease, affects approximately 1% of the global population [2]. The primary pathological features of RA include persistent synovitis and joint bone erosion [3], which occur due to a continuous influx of immune cells into the joints and their interactions with osteocytes, particularly osteoclasts.

Within this context, effector T cells, along with B cells and other innate effector cells, form a complex network that circulates to amplify the inflammatory response. These cells secrete several inflammatory factors such as IL-17, IFN-γ, IL-6, TNF-α, and IL-1β. These factors can activate fibroblast-like synoviocytes (FLS), which are tissue-resident mesenchymal stromal cells in the joints. The activation of FLS leads to tissue destruction and the production of additional inflammatory factors [3]. FLS, in conjunction with other target cells supporting osteoclastogenesis, express receptor activator of nuclear factor-κB (NF-κB) ligand (RANKL), which triggers the development and functional maturation of osteoclasts. This process promotes bone destruction and the expression of matrix metalloproteinases (MMPs), accelerating cartilage degradation [4]. Importantly, osteoclasts play a significant role in local bone erosion, periarticular osteopenia, and systemic osteoporosis in RA.

The current therapies available for managing bone destruction in patients with RA are quite limited, and not all patients respond well to existing treatment options. Thus, it is crucial to develop new targeted drugs that specifically address the key cells involved in bone loss, such as osteoclasts, T cells, and B cells. By unraveling the immune mechanisms underlying joint destruction in RA, we can establish a solid scientific foundation for innovative therapeutic approaches.

In this context, it is essential to recognize the significant interplay between the immune system and osteoclasts. Specifically, the interaction and crosstalk between T cells, B cells, and osteoclasts play a pivotal role. This article provides a comprehensive review of recent advancements in these areas, highlighting the intricate communication between T cells, B cells, and osteoclasts in the immune mechanisms of RA. Additionally, it summarizes the promoting or inhibitory effects of various subtypes of T and B cells, as well as the inflammatory factors they secrete, on osteoclasts in the context of RA. Additionally, the report examines novel biomarkers for RA and existing drugs that target osteoclasts, either directly or indirectly, to address RA-related bone erosion. The report also provides valuable insights into potential therapeutic approaches for RA in the future.

## 2. The Crosstalk between T Cells, B Cells, and Osteoclasts in the Immune Machinery of RA

**Figure 1:**
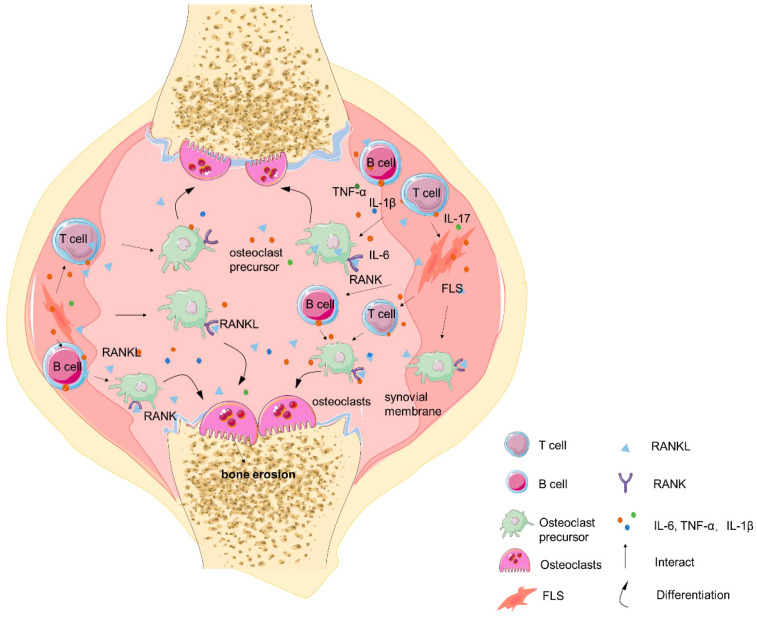
T cells, B cells, and osteoclasts in the immune machinery in RA. This figure illustrates the role of T cells, B cells, and osteoclasts in the immune machinery of RA. Activated T cells and B cells secrete pro-inflammatory factors such as IL-1β, IL-6, and TNF-α, among others. In the inflammatory microenvironment of RA joints, these cytokines promote the differentiation of FLS into tissue-damaging FLS and the release of RANKL—the primary factor promoting the generation of osteoclasts. Furthermore, T cells and B cells also contribute to the secretion of RANKL. Upon the junction of RANKL with RANK on the osteoclast precursors, a cascade of pathways will induce the differentiation of osteoclasts, leading to their maturation and ultimately contributing to bone erosion.

RA is a chronic autoimmune disease characterized by inflammation of the synovial tissue and progressive destruction of cartilage and bone, primarily caused by excessive activity of osteoclasts, leading to abnormal bone erosion [5]. In the context of RA, both T cells and B cells play crucial roles as key mediators of the immune response, with T cells being major contributors to cellular immunity and B cells involved in humoral immunity [6]. The interaction between osteoclasts, T cells, and B cells further aggravates the disease, although the precise immunopathogenesis of RA remains unclear. Figure 1 illustrates the general relationship between the interactions of T cells, B cells, and osteoclasts within the RA joint.

T cells play a crucial role in maintaining basal bone turnover and regulating peak bone mass. This has been confirmed by studies showing that T cell-deficient nude mice exhibit osteoporosis and reduced production of osteoprotegerin (OPG) [3]. However, under pathological conditions, activated T cells can disrupt bone homeostasis and contribute to bone destruction [3]. In RA, the immune response is initiated by T cells recognizing antigens presented by antigen-presenting cells, such as dendritic cells (DCs). CD4^+^ T cells then differentiate into helper T cells (Th), with Th17 cells playing a key role in RA inflammation [7]. An imbalance between Th17 and regulatory T (Treg) cell commitment has been identified in RA. While Th17 cells promote inflammation by secreting IL-17 and IL-22, Tregs suppress hyperinflammation by mediating immune response suppression and immune tolerance through the production of IL-10 and/or TGF-β [8,9]. Although there is some conflicting data, the majority of studies have shown a reduced proportion of circulating Tregs in RA. Moreover, the infiltration of CD4^+^ Th cells at sites of inflammation is a characteristic feature of autoimmune diseases and RA. High frequencies of citrullinated-specific Th1 cells have also been observed in patients with RA [10]. Additionally, T cells serve as an important source of RANKL and TNF-α in RA [3]. The proliferation of fibroblast-like FLS and innate immune cells like neutrophils and macrophages, as well as the expression of proinflammatory cytokines (e.g., TNF-α, IL-6, and IL-1β) and chemokines (e.g., CCL20 and CCL2), are mediated by IL-17 and other cytokines (e.g., IL-21, IL-22, and TNF-α) [7]. FLS generates mechanical strains, further amplifying the inflammatory effects of these mediators [11]. A 2019 study using single-cell RNA sequencing (scRNA-seq) indicated that FLS are the main producers of IL-6, while macrophages are the main producers of IL-1β and TNF-α. T and B cells have also been found to produce TNF-α in RA [12].

The activated T cells can facilitate the production of autoantibodies, such as anti-citrullinated peptide antibodies (ACPAs) and rheumatoid factor (RF), by B cells. ACPAs have been shown to directly promote osteoclastogenesis both in vitro and in vivo [13]. Basal-conditioned B cells are important producers of OPG under normal physiological conditions. Studies have shown that B cell-deficient mice displayed increased osteoclast formation and bone resorption, and both the OPG protein and mRNA levels were significantly reduced in bone marrow cells (BMs) with B cell knockout (KO). However, when the B cells were transplanted back into B cell KO mice, OPG levels were completely normalized. In contrast, total quantities of BM RANKL mRNA were not significantly affected by B cell depletion [14]. These data support the idea that basal-conditioned B cells can regulate their production either by secreting OPG or indirectly and are not an important source of RANKL. Nevertheless, depending on the microenvironment in which the B cell is located, different transcription products are produced, and different effector proteins are secreted. For instance, under inflammatory conditions, B cells are converted to RANKL producers in addition to ACPAs and RFs [15]. It seems that B cells play an important role in regulating the generation of osteoclasts through the RANKL/OPG signaling system. Interestingly, B cells even undergo further differentiation into osteoclasts in the presence of macrophage colony-stimulating factor (M-CSF) and RANKL during in vitro co-culture [16]. Research has shown that B cell depletion therapy (with rituximab) significantly reduces clinical symptoms and inflammation in RA, as well as inhibits the progression of structural joint damage by increasing bone formation and decreasing bone resorption [15,17]. These findings emphasize a link between B cells and bone homeostasis in RA and suggest that B cells may play a key pathogenic role in RA bone erosion. However, the effects of B cells on osteoblasts have been less well-studied, although there are indications that B cells may inhibit osteoblast differentiation through CCL3 and TNF-α signaling [18]. Taken together, these lines of evidence suggest that B cells are not absolute protectors or destroyers of bone erosion.

## 3. Regulatory Signals for Osteoclast Differentiation and Maturation

Osteoclasts are specialized, terminally differentiated cells derived from the hematopoietic monocyte–macrophage system. They are stimulated by cytokines such as M-CSF and RANKL. Initially, osteoclast precursors differentiate into TRAP-positive monocytes, which then migrate and attach to sites such as bone surfaces and around intraosseous vascular channels. Subsequently, they transform into giant multinucleated cells through intercellular fusion and incomplete cell division, thus enabling them to degrade (resorb) the skeletal matrix by generating a large sealing zone consisting of an actin ring containing fold boundaries [19]. However, the maturation of osteoclasts is influenced by a number of factors, including both positive and negative regulators.

### 3.1. Migration of Osteoclasts

Recruitment of osteoclast precursors to the endosteum of bone with high expression of RANKL is a critical step in osteoclast differentiation. Among these precursors, CXCL12, which is highly expressed in specific stromal cells enriched in the perivascular region of the bone marrow cavity, has been shown to promote the chemotactic recruitment, development, and survival of osteoclast precursors that abundantly express CXCR4 [20]. Another chemokine, CX3CL1 (fractalkine), has also been demonstrated to be involved in the migration of osteoclast precursors. While CX3CL1 is expressed by osteoblasts, osteoclast precursors preferentially express its cognate receptor, CX3CR1. CX3CL1 has been found to play an important role in the recruitment of osteoclast precursors to the bone marrow lumen as well as in their firm adhesion to the endothelium [21,22].

Sphingosine-1-phosphate (S1P), similar to chemokines, plays a crucial role in regulating lymphocyte chemotaxis. osteoclast precursors express two receptors for S1P, namely S1PR1 and S1PR2, both of which regulate the migration of osteoclast precursors. In blood vessels with high concentrations of S1P, the activity of S1PR2 dominates, leading to the entry of osteoclast precursors into the bone cavity through S1PR2-mediated chemotaxis. However, when osteoclast precursors enter the bone marrow with low S1P levels, S1PR1 is reactivated, allowing some osteoclast precursors to return to the vasculature following the S1P gradient. The balance between osteoclast precursors entering and leaving circulation governs the amount of osteoclast precursors on the bone surface [23,24]. This newly discovered homeostatic factor contributes to osteoclastogenesis in vivo.

### 3.2. Positive Regulation of Osteoclast Differentiation and Maturation

**Figure 2:**
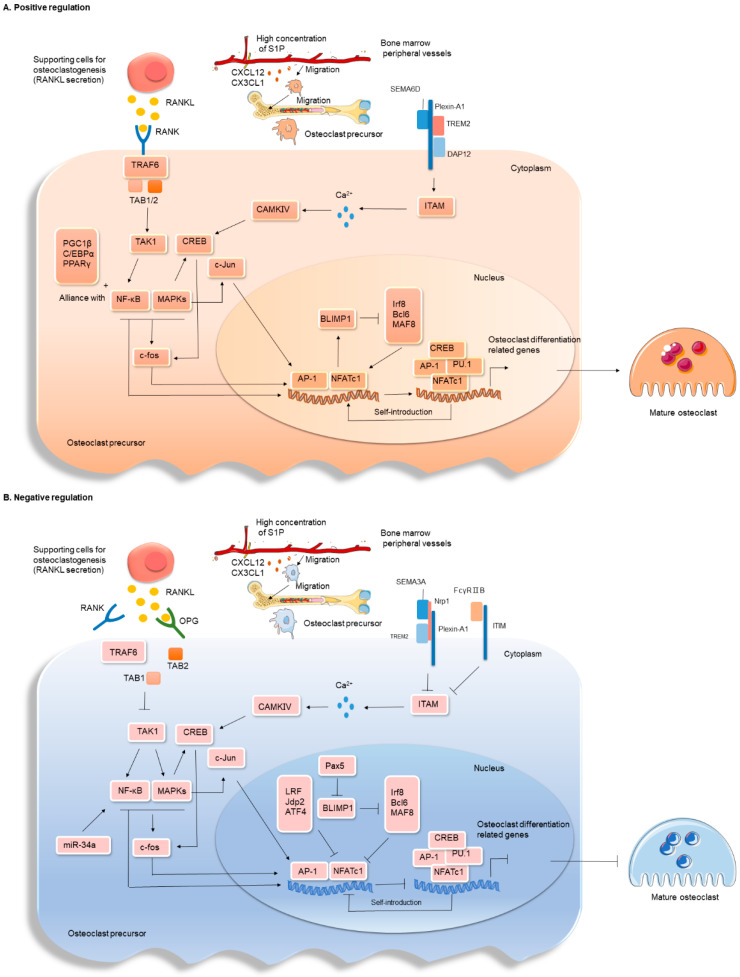
Positive and negative regulation of osteoclast differentiation. Initially, osteoclast precursors are located in the periosteal vasculature due to high concentrations of S1P. Subsequently, they migrate into the bone cavity in response to varying S1P concentrations and chemokines such as CXCL12 and CX3CL1. Upon reaching their destination, the osteoclast precursors differentiate and mature under the influence of pro-osteoclast genesis factors, including RANKL. (**A**) RANKL binds to RANK receptors expressed by osteoclast precursors, forming complexes with TRAF6, TAB1, and TAB2. This interaction activates TAK1 and triggers MAPKs and NF-κB cascade signaling. Consequently, activated c-Fos induces the expression of NFATc1. NFATc1 then binds to AP-1, CREB, PU.1, and other targets, including its own promoter, leading to the transcription of various osteoclast-specific genes (such as Ctsk, MMP9, etc.), creating a self-induced closed loop. NFATc1 also induces the transcriptional repressor BLIMP1, which inhibits the generation of anti-osteoclast genes like Irf8, Bcl6, and Mafb. CaMKIV-CREB signaling, PPARγ, PGC1β, and C/EBPα signaling can synergize with NF-κB signaling to induce c-Fos and consequently promote NFATc1 expression. Additionally, SEMA6D enhances osteoclast formation by activating ITAM signaling, followed by CaMKIV-CREB signaling, through the formation of the Plexin-A1-TREM2-dap12 complex. (**B**) OPG competes with RANKL for binding to RANK, thereby blocking the signals triggered by RANKL in the positive regulatory pathway described above. Notably, Irf8 and Bcl6 directly interact with and inhibit the expression of NFATc1. Furthermore, other transcription factors including ATF4, LRF, and Jdp2 negatively regulate NFATc1. Pax5 inhibits BLIMP1 expression, FcγRIIB inhibits ITAM signaling, and the SEMA3A-nrp1 axis restricts ITAM signaling by segregating Plexin-A1 from TREM2. Additionally, miR-34a acts as a negative regulator of osteoclast differentiation by inhibiting NF-κB signaling. Another receptor for RANKL, lGR4, functions similarly to OPG in blocking RANKL signals.

Once a suitable environment for osteoclastogenesis is reached, the next critical step is differentiation and maturation. Among the many regulators that promote osteoclast differentiation and maturation, the most important is RANKL expressed by various cells including FLS, osteoblasts, osteocytes, and T cells. When RANKL binds to its receptor, RANK, expressed by osteoclast precursors, it forms a complex with TRAF6, TAB1, and TAB2 to activate TAK1, which in turn triggers the activation and amplification of MAPKs and NF-κB cascade signaling [25,26]. Subsequently, c-Fos activation induces the expression of another major transcription factor for osteoclastogenesis, the nuclear factor of activated T cells 1 (NFATc1). Interestingly, NFATc1 can synergistically induce the transcription of a variety of osteoclast-specific genes, such as Ctsk and Mmp9, in combination with its own promoter, as well as other transcription factors, such as AP-1, CREB, and PU.1, thus forming a closed loop of amplification that promotes osteoclast differentiation and maturation [25,27]. B lymphocyte-induced maturation protein 1 (BLIMP1) acts as a transcriptional repressor of anti-osteoclastogenic genes, such as interferon regulatory factor 8 (Irf8), B cell lymphoma 6 (Bcl6), and MAF bZIP transcription factor B (Mafb), and is also a target gene for initiation of NFATc1 expression [25]. Additionally, calcium-/calmodulin-dependent protein kinase IV (CaMKIV)-CAMP-response element binding protein (CREB) signaling, peroxisome proliferators-activated receptors γ (PPARγ), peroxisome proliferator-activated receptor gamma co-activator 1β (PGC-1β), and CCATT enhancer-binding protein α (C/EBPα) signaling pathways synergize with NF-κB signaling for the induction of c-Fos, which is defined as an inducer of NFATc1 [28]. Signaling proteins, which play key roles in the immune and nervous systems in controlling osteoclast formation, are axon guidance molecules. Semaphorin 6D (SEMA6D) promotes osteoclast formation by initiating the Plexin-A1-TREM2-dap12 complex via activation of ITAM signaling and subsequent CaMKIV-CREB signaling [25,29] (Figure 2).

### 3.3. Negative Regulation of Osteoclast Differentiation and Maturation

An established inhibitor of osteoclastogenesis is OPG, a competitive ligand for RANKL that diminishes the binding of RANKL to RANK and hinders the generation of osteoclasts by interrupting the signaling that promotes their formation [30]. Inhibiting pro-osteoclastogenic transcription factors is also crucial for effectively preventing osteoclastogenesis. As mentioned earlier, Irf8 and Bcl6 can be inhibited by directly interacting with and repressing NFATc1 expression, respectively [25]. Other transcription factors such as activating transcription factor 4 (ATF4), locoregional failure (LRF), and Jun dimerization protein 2 (Jdp2) have also been reported to play a role in negatively regulating NFATc1. Intriguingly, LRF negatively regulates osteoclast differentiation by repressing NFATc1 induction in the early phase of osteoclast development, while positively regulating osteoclast-specific genes by acting as a coactivator of NFATc1 during the bone resorption phase [31,32,33]. Additionally, paired box protein 5 (Pax5) inhibits BLIMP1 expression and reduces the inhibition of anti-osteoclastogenic factors to alleviate osteoclastogenesis [34]. FcγRIIB and FcγRⅢ are highly expressed in osteoclast precursors [35]. FcγRIIB inhibits osteoclastogenesis by suppressing ITAM signaling, and the SEMA3A-nrp1 axis inhibits osteoclast formation by isolating Plexin-A1 (a receptor shared by SEMA3A and SEMA6D) from TREM2 [25,35]. Conversely, FcγRⅢ blocks osteoclastogenesis by sequestering FcRγ from OSCAR and PIR-A [35]. The microRNA miR-34a inhibits osteoclastogenesis by repressing Tgif2, a negative regulator of osteoclast differentiation involved in JNK and NF-κB signaling in osteoclasts [36]. Another receptor for RANKL, LGR4, has been reported to negatively regulate osteoclast formation by competing for RANK binding with RANK and inhibiting NFATc1 activation through G-αq [37]. Furthermore, a mutation in the Ptpn6 gene encoding SHP-1, a nonreceptor tyrosine-protein phosphatase, leads to low bone mass due to an increase in osteoclasts number [38,39]. The ecological niche space formed by osteoclast bone resorption is essential for normal hematopoiesis. The inhibitory effect of anti-resorptive drugs on osteoclast activity affects hematopoietic stem cells and immune cells. For instance, in wild-type (WT) mice, bisphosphonate administration reduced the frequency and number of hematopoietic stem cells and B cells [40,41]. Osteoclast precursors have been reported to possess the ability to inhibit T cell proliferation in a mouse model of autoimmune arthritis [25]. From the regulatory network of osteoclastogenesis, it is evident that many regulatory factors are shared with the immune system, where the binding of RANKL to RANK appears to be the starting point of osteoclastogenesis and an effective bridge connecting the communication network of osteoclasts and immune cells. Therefore, it is crucial to further understand the shared regulatory mechanisms between RANKL and immune cells (Figure 2).

## 4. Regulation of T Cells by RANKL

RANKL belongs to the TNF cytokine family. Despite its primary role in bone remodeling, RANKL has been found to regulate immune responses and the development of immune organs, similar to other pleiotropic cytokines of the TNF superfamily [42,43]. T cells are capable of producing RANKL, which leads to their activation and subsequently inducing T cell signaling, proliferation, and viability through IL-4 and transforming growth factor. Mice with disrupted RANKL genes exhibited dysfunctional early T cell differentiation and development, although their spleen structure and dendritic cell development remained normal [44]. In IL-2 deficient mice, high expression of RANKL in T cells resulted in intestinal CD11c^+^ DCs survival, leading to bone loss and colitis [45]. Furthermore, the transfer of DCs stimulated by RANKL exacerbated autoimmunity in MRL/lpr-recipient mice [38]. Under specific experimental conditions, the pro-inflammatory effects of RANKL on DCs may be particularly emphasized. In CD40-deficient mice, RANK-Fc treatment was able to suppress T cell-dependent immune response and thus suppress viruses and parasites, suggesting that CD40L signaling compensated for the absence of RANKL signaling [38]. RANKL inhibitors not only effectively inhibit bone loss but also promote host inflammatory responses by interfering with Treg activity, indicating an immunomodulatory role for this classic osteoclast mediator [42]. Blocking RANKL signaling by RANK-Fc proteins led to a reduction in Treg cell numbers, resulting in a worsening of the type 1 diabetes model [46]. However, after the withdrawal of the anti-RANKL drug, a modest increase in the number of Treg cells was observed, suggesting that the changes in immunomodulation may be reversible and continue for a longer period than the rapid reversible effects observed in bone. Therefore, the effect of RANKL on Tregs may be a feedback mechanism for immunomodulation [47].

## 5. Regulation of B Cells by RANKL

Both B cell maturation and differentiation occur in the bone marrow, which is in close proximity to osteoblasts and, thus, the two have close communication and connection. Cytokines that affect bone metabolism, such as TNF-α, IL-1β, and IL-13, as well as vascular cell adhesion molecules also directly influence B cell homing and differentiation [48]. Germline deletion of *Tnfrsf11a* (encoding RANK) or *Tnfsf11* (encoding RANKL) genes results in impaired B cell development. Moreover, *TNFSF11A* mutations lead to a decrease in human immunoglobulin-secreting B cells [38]. Inhibition of systemic RANKL-RANK signaling may lead to severe osteoporosis resulting in the loss of bone marrow microenvironment for B cell development. Marrow B cell development was also impaired in Rag1-deficient mice reconstituted with fetal hepatocytes from *Tnfsf11*-deficient mice, suggesting the importance of RANKL for B cell development in hematopoietic lineage cells. However, conditional deletion of the *Tnfrsf11a* gene in B cells had no significant effect on B cell development or antibody production, indicating that RANK on B cells is unnecessary for B cell development and antibody production [49].

## 6. Crosstalk between T Cells and Osteoclasts in RA

### 6.1. Crosstalk between Th17/Treg and Osteoclasts

Th17 and Treg cells play immune-activating and immune-suppressing roles, respectively. Th17 cells and their effector molecule IL-17A are involved in various effector functions associated with RA pathology, and Th17 is also one of the major cell subpopulations supporting differentiation of osteoclasts during osteoclastogenesis, which is induced by RANKL and carried in combination with RANK on osteoclast precursors. IL-17A indirectly stimulates cells that support the generation of osteoclasts, such as fibroblast-like FLS and osteoblasts, to express RANKL and increase bone erosion [50,51]. Sato et al. demonstrated that IL-17A is essential for bone destruction in a mouse model of RA [52]. IL-17A expression is significantly increased in RA, with Th17 cells isolated from RA joints expressing higher levels of RANKL; moreover, Th17 cells can induce the production of inflammatory factors such as TNF-α, IL-1β, and IL-6, and have the ability to induce the expression of RANKL on FLS, indirectly contributing to the generation of osteoclasts [50,51]. Fixed-activated T cells were reported to act directly on osteoclast precursors shortly after RANKL cloning and to induce osteoclastogenesis by RANKL acting on T cells in vitro [53]. However, live T cells do not have this osteoclastogenic capacity because they produce IFN-γ, which effectively inhibits osteoclast differentiation [54].

Treg cells have been categorized into two main groups: naturally occurring Treg cells (nTregs) and induced Treg cells (iTregs) [55]. The nTregs are naturally found in the thymus, while iTregs are produced by naïve T cells in peripheral lymphoid tissues in response to autoantigen stimulation [55]. Tregs demonstrate anti-inflammatory effects by inhibiting and regulating the activity of inflammatory T cells to maintain peripheral tolerance, and also promote apoptosis of osteoclast precursors to inhibit bone resorption [56]. Studies have revealed that the main mechanisms through which Treg cells affect bone include cell contact-dependent and suppressor cytokine mechanisms. Among them, Treg cells expressing cytotoxic T-lymphocyte-associated antigen-4 (CTLA-4) bind to CD80/CD86 on the surface of osteoclast precursors, inducing the activation of indoleamine-2,3-dioxygenase in the osteoclast precursors, thereby promoting apoptosis of the osteoclast precursors to inhibit bone resorption, which constitutes the cell contact-dependent mechanism [57,58]. Additionally, Treg cells inhibit osteoclast production by secreting cytokines such as TGF-β and IL-10, representing the inhibitory cytokine mechanism [59]. Furthermore, it has been observed that the inhibitory effect of Treg cells on osteoclast differentiation is enhanced in the presence of estrogen [60]. This suggests that estrogen may act as a synergistic inhibitor of osteoclasts inhibition by Treg cells, or could estrogen act as a stimulator of Treg cells, enhancing the strength of Treg action or increasing the number of Treg cells? This requires further study.

Interestingly, there is a high level of flexibility in the specific roles of Th17 and Treg cells, as they can switch functions depending on the cytokine environment they are exposed to [61]. For instance, when exposed to increased concentrations of cytokines produced by Th17 cells, Treg cells can also secrete IL-17, indicating their inherent instability. In RA, a subset of Treg cells known as exFoxp3 Th17 cells has been identified. These cells have lost the ability to express and regulate Foxp3 but gained the ability to produce Th17-associated cytokines such as IL-17A and RANKL. Notably, exFoxp3 Th17 cells exhibit greater osteoclastogenic capacity compared to regular Th17 cells [62,63]. Furthermore, these cells upregulate the expression of the senescence marker killer cell lectin-like receptor G1 (KLRG1). KLRG1^+^ Treg cells show reduced proliferative capacity and inhibitory function in vitro and in vivo, adopting a phenotype and function characteristic of Th17 cells [64]. Both nTregs and iTregs can be converted into Th17 cells in the presence of IL-6 and TGF-β or IL-1 and IL-23, as demonstrated by Yang et al. Additionally, Foxp3/IL-17 double-positive T cells play a role as intermediary cells in the conversion from Th17 cells to Treg cells. If the balance tilts toward inflammatory Th17 cells, it may lead to an increase in osteoclasts and promote bone resorption [65,66].

Given the dynamic interplay between these two cell types, maintaining a balance is crucial in preventing bone erosion in osteoimmunopathies. This balance also holds promise as a potential therapeutic target.

### 6.2. Crosstalk between Young T Cells/Senescent T Cells and Osteoclasts

Cellular senescence is a gradual decline in cell proliferation, differentiation capacity, and physiological function over time. Immunosenescence, on the other hand, refers to the senescence of immune cells within the immune system, including CD4^+^ T cells. The primary marker used to identify senescent T cells is the loss of CD28, a co-stimulatory receptor responsible for antigen presentation and effective T cell activation [67,68]. Senescence is characterized by telomere erosion, increased expression of pro-inflammatory molecules, and impaired effector functions. In various diseases such as RA, juvenile idiopathic arthritis, osteomyelitis, and osteoporosis, there is often an elevated presence of prematurely senescent CD4^+^CD28^−^ T cells [69,70,71].

It has been demonstrated that RA patients with concurrent bone loss have the highest levels of CD4^+^CD28^−^ T cells when compared to patients with osteoporosis and those with normal bone mass. In the non-RA group, researchers also noted an increased percentage of CD4^+^CD28^−^ T cells and CD8^+^CD28^−^ T cells in patients with reduced bone mass. While lumbar vertebral bone mineral density (BMD) did not show a correlation with CD4^+^CD28^−^ T cells in the general cohort, it was found to have a significant correlation in the RA cohort. Additionally, CD4^+^CD28^−^ T cells were found to produce higher levels of osteoclastic molecular mediators, such as TNF-α, IL-17A, and RANKL, which are directly associated with pathological bone loss when compared to normal CD4^+^CD28^+^ T cells. Furthermore, more TRAP^+^ multinucleated cells and larger areas of bone resorption were observed after replacing CD4^+^CD28^+^ T cells with CD4^+^CD28^−^ T cells when compared to the co-culture system of monocytes and CD4^+^CD28^+^ T cells [71]. Furthermore, CD8^+^CD28^−^ T cells have been found to have a negative correlation with bone healing and regeneration, as they inhibit osteoblast differentiation and mesenchymal cell survival [67,72]. Additionally, premature senescence of T lymphocytes directly increases Th17 cell activity. The detrimental effects of CD4^+^CD28^−^ T cells on bone are thought to be associated with the functional imbalance between Th17 and Treg cells, as well as the phenotypic instability of Treg cells. These Treg cells can undergo transdifferentiation into RANKL-producing exFoxp3 Th17 cells, as mentioned earlier [73,74]. In RA patients, it has been reported that there is an increased number of Foxp3^+^CD4^+^CD28^−^ T cells compared to Foxp3^+^CD4^+^CD28^+^ cells. These senescent Treg cells exhibit lower levels of CD25 expression, higher cytosolic senescent β-galactosidase activity, and produce higher levels of TNF-α, IFN-γ, and IL-17A. Senescent Treg cells that lose CD28 share similarities with exFoxp3 Th17 cells, as CD28 plays a direct role in Foxp3 expression and maintenance of high levels of CD25. As a result, exFoxp3 Th17 lymphocytes may lose the ability to regulate Foxp3 and CD25 due to the lack of CD28 co-stimulation. This further favors Th17/Treg imbalance, leading to increased production of pro-inflammatory senescence-associated secretory phenotype-associated cytokines, as well as inducing osteoclast production and pathological bone resorption [67,75,76].

The available evidence strongly supports the significant contribution of senescent T cells to bone diseases characterized by excessive osteoclast activity. Consequently, preventing T cell senescence appears to be a promising approach to mitigating bone diseases caused by osteoclast overactivity.

## 7. Crosstalk between B Cells and Osteoclasts in RA

### 7.1. Crosstalk between Memory B Cells and Osteoclasts

Memory B cells, a type of B lymphocyte, retain information about a pathogen and can rapidly produce the appropriate antibodies upon reinfection by the same pathogen. Activated memory B cells exhibit a high potential to produce RANKL. Studies have demonstrated the enrichment of memory B cells in the synovial microenvironment of RA, particularly in the synovial tissue of RA patients undergoing elective or arthroscopic orthopedic surgery. This subpopulation is distinguished by its high spontaneous expression of CD95 and its ability to produce more RANKL than T cells [15]. Furthermore, there is a significant expansion of CD95^+^-activated memory B cells in the peripheral blood of RA patients, which can spontaneously express RANKL [77].

### 7.2. Crosstalk between Regulatory B Cells and Osteoclasts

Given the significant role of IL-10-producing B cells in immune modulation, a specific subset of regulatory B cells (Bregs) known as “B10 cells” was defined by LeBien and Tedder [78]. These Bregs exhibit anti-inflammatory potential primarily through the production of IL-10 cytokines, and their therapeutic efficacy has been demonstrated in various disease models, including cancer, autoimmune diseases, and infectious diseases. By producing IL-10, IL-35, and TGF-β, B10 cells exert immunosuppressive effects by inhibiting the expansion of diverse immune cells, including T lymphocytes, while promoting Treg differentiation [79]. However, the precise role of Bregs in regulating osteoclast generation has yet to be fully elucidated. In one study conducted under in vitro conditions, the anti-osteoclastogenic potential of Bregs was explored, revealing their ability to dose-dependently inhibit osteoclast differentiation induced by RANKL [80]. Multiple studies in both humans and mice have highlighted that Bregs exert inhibitory effects on inflammatory responses through the production of IL-10 cytokines [81]. Moreover, it has been further established that the anti-osteoclastogenic properties of Bregs are mediated through their production of IL-10 [80].

Furthermore, IL-10-producing Bregs play a crucial role in maintaining tolerance in response to signals derived from the gut microbiota. Recent studies have highlighted the potential involvement of gut microbiota dysbiosis in the pathogenesis of RA [82]. However, the mechanism through which microbiota dysbiosis leads to abnormal immune cell function in human arthritic disease remains elusive. Previous reports have indicated an inverse correlation between the frequency of CD19^+^CD24^hi^CD38^hi^ B cells, which harbor the highest proportion of IL-10^+^ Bregs, and the clinical severity of RA [83]. The inflammatory signaling in gut-associated lymphoid tissue is believed to provide a milieu conducive to the differentiation of immature B cells into Bregs, due to the interplay between the gut microbiota and the innate immune system [84]. Notably, studies involving mice depleted of endogenous bacteria after receiving broad-spectrum antibiotics have suggested that the microbiota plays a significant role in both inflammation development and Breg differentiation, although whether it is an essential condition remains unknown. As of now, there is no definitive answer regarding the sequential, primary, or secondary relationship among these factors.

Interestingly, both RA patients and arthritic mice exhibited lower levels of the microbial-derived short-chain fatty acid (SCFA) butyrate in their feces, a phenomenon associated with reduced frequencies of CD19^+^CD24^hi^CD38^hi^ B cells and IL-10^+^ Bregs. Notably, in mice, supplementation with SCFA butyrate was shown to decrease the severity of arthritis. This effect may be attributed to the elevation of serum-derived metabolite 5-hydroxyindole-3-acetic acid, which activates the aryl hydrocarbon receptor, a newly identified transcriptional marker for Bregs, leading to the suppression of arthritis in a Breg-dependent manner [83]. Consequently, this study suggests that butyrate supplementation could serve as a promising therapy for alleviating systemic autoimmune diseases.

### 7.3. Crosstalk between Plasma Cells and Osteoclasts

Plasma cells, which synthesize and store antibodies, known as immunoglobulins, and participate in the humoral immune response, are also known as effector B cells. In the bone marrow of arthritic mice, RANKL-expressing plasma cells are increased in number and show the ability to induce osteoclast differentiation in vitro [85]. Plasma cells promote generation and periarticular bone erosion in autoimmune arthritis [85]. *Fra1* controls the production of RF autoantibodies in bone marrow plasma cells and the development of autoimmune bone loss. Deficiency of *Fra1* in B cells leads to an increase in IgG1-producing bone marrow plasma cells, enhanced production of IgG-RF, and an increase in bone loss associated with elevated osteoclast counts after immunization. The effect of IgG-RF on osteoclasts in vitro and on osteocytes associated with bone loss in vivo is dependent on FcγR, particularly FcγR3 [56]. The effects of IgG-RF were dependent on FcγR, particularly FcγR3. It has been reported that B cells and B cell-derived plasma cells in multiple myeloma support osteoclast production possibly through direct expression of RANKL or as an indirect result of secretion of IL-7, a potent stimulator of bone resorption in vivo [86,87].

### 7.4. Crosstalk between B Cell Exosomes and Osteoclasts

Exosome secretion by B cells is considered one of the mechanisms through which B cells exert their actions, and it has been implicated in bone diseases. To investigate the role of B cell-derived exosomes (BC-Exos) in bone homeostasis in vitro, researchers conducted an experiment in which mouse bone marrow mononuclear cells were treated with BC-Exos or an equal volume of PBS. The results demonstrated a direct correlation between the concentration of BC-Exos and the degree of osteoclast differentiation. Furthermore, the expression levels of Ctsk and DC-STAMP mRNA, which serve as marker genes for osteoclast differentiation, were significantly upregulated on day 7 of BC-Exos treatment compared to the control group. Additionally, pre-osteogenic MC3T3-E1 cells were also subjected to in vitro treatment with BC-Exos or an equal volume of PBS. The BC-Exos-treated MC3T3-E1 cells exhibited reduced mineral deposition, and the expression levels of osteoblasts maturation marker genes, namely BGLAP and alkaline phosphatase (*Alp*) mRNA, were significantly decreased on day 21 of the experiment.

Collectively, these findings suggest that BC-Exos acts as an inhibitor of osteoblast differentiation and a promoter of osteoclast differentiation in vitro. To further elucidate the effects of BC-Exos on osteoblasts and osteoclasts in vivo, BC-Exos or an equal volume of PBS was administered via injection into the tail vein of 8-week-old male mice. Micro-computed tomography (m-CT) results revealed a significant reduction in both the volume and number of bone trabeculae, as well as a notable increase in trabecular separation in mice treated with BC-Exos compared to the control group [88].

In conclusion, BC-Exos plays a role in bone homeostasis by promoting bone resorption in osteoclasts and inhibiting bone formation in osteoblasts. Despite this understanding, there is a scarcity of studies examining the effects of BC-Exos on osteoclasts and osteoblasts, highlighting the need for additional research in this area. Further exploration of the components and classes of BC-Exos could provide valuable insights to help us develop strategies aimed at reducing BC-Exos production and release, ultimately leading to improvements in bone homeostasis.

## 8. Effect of Related Cytokines on Osteoclasts

### 8.1. Effects of TNF-α on Osteoclasts

The available evidence indicates that TNF-α contributes to bone loss in addition to initiating chronic inflammation. The use of TNF-α inhibitors has been shown to reduce the formation of osteoclasts and may alleviate inflammation and bone erosion in RA. Mechanistically, TNF-α promotes osteoclasts by increasing the expression of RANK and its ligand RANKL [89,90]. In animal models, TNF-α induces osteoporosis by upregulating RANK expression and reducing OPG production, thereby promoting osteoclast differentiation [91]. Interestingly, studies have revealed that in mice without osteoclasts, TNF-α does not lead to bone loss [92].

The reduction in osteoclast production following TNF-α inhibitor treatment is believed to be primarily attributed to a decrease in RANKL expression, rather than TNF-α itself [90]. Previous studies have indicated that non-responsive patients with RA often exhibit higher levels of Th17 cells, and the persistence of disease in TNF-α inhibitor-treated patients may be driven by IL-17 [93]. Additionally, TNF-α inhibits osteoblasts and promotes their apoptosis by increasing the expression of dickkopf-related protein 1, a Wnt antagonist that can negatively impact new bone formation [90,94]. Studies on osteoporosis in elderly individuals have further supported the role of TNF-α in systemic osteoporosis [95].

In skeletal RA, bone loss initiates early and progresses rapidly. If the disease is not effectively controlled, the BMD of the vertebral body and femoral neck can decrease by 2.5% within the first year and double in the second year [96]. Evidence suggests that TNF-α inhibitors can reduce inflammation and mitigate bone erosion in RA [97]. However, it remains unclear whether bone loss improves in the absence of a clinical response, as approximately 50% of RA patients receiving TNF-α inhibitors do not respond to treatment or experience relapse after an initial positive response. Studies investigating bone loss have shown that TNF-α inhibitors have the potential to reduce osteoclast formation and alleviate inflammation and bone erosion in RA. Interestingly, in mice without osteoclasts, TNF-α did not induce bone loss. It is believed that the decrease in osteoclast production following TNF-α inhibitor treatment is primarily attributed to reduced expression of RANKL, rather than TNF-α itself [90]. Therefore, comprehending the mode and characteristics of TNF-α’s impact on osteoclasts and the precise mechanism of action in RA is crucial for finding ways to reduce bone erosion in this condition.

### 8.2. Effects of IL-7 on Osteoclasts

IL-7 is a cytokine produced by bone marrow cells, spleen, and thymus stromal cells, and it plays a crucial role in the development of B cells. Signaling through the IL-7 receptor (IL-7R) triggers the proliferation of B cell progenitors. Studies involving mice with targeted deletion of IL-7 or IL-7R have demonstrated a significant impairment in early pro-B cell development [98,99]. Interestingly, despite the lack of IL-7 or IL-7R, these mice were still able to detect the presence of B cells in the periphery, indicating that IL-7 or IL-7R is not essential for B lymphocyte production [100].

A study has reported that IL-7 can directly induce osteoclastogenesis by activating STAT5 in a RANKL-independent manner [101]. Additionally, IL-7 has been found to have potent osteolytic effects in mouse models by upregulating RANKL on T cells [102]. Moreover, mice with global overexpression of IL-7 exhibited reduced bone mass and increased osteoclasts, while osteoblasts remained unchanged [103]. However, there are also reports indicating that IL-7 may function as a potential inhibitor of osteoclastogenesis in vitro [104]. As of yet, the exact mechanism of IL-7 in bone homeostasis has not been clearly elucidated.

### 8.3. Effects of IL-6 on Osteoclasts

Abnormally elevated levels of IL-6 have been found in various chronic injuries and inflammatory diseases. IL-6 is known to promote RANKL and inhibit OPG, thus being associated with osteolysis, osteoporosis, RA, and other bone-related conditions [15,105,106]. Moreover, IL-6 reduces the differentiation of osteoblasts by downregulating the expression of genes involved in osteoblast differentiation, such as *Alp*, *Runx2*, and *osteocalcin*. The IL-6/sIL-6R signaling pathway also impairs the ability of osteoblasts to mineralize bone through the mitogen-activated extracellular signal-regulated kinase/extracellular signal-regulated kinase and phosphoinositide 3-kinase/serine/threonine kinase AKT2 pathways. Inhibition of these pathways can enhance the expression of Runx2 and other mature osteoblast phenotypes [107]. Additionally, studies have reported that IL-6, when co-stimulated with TNF-α, induces the differentiation of bone marrow-derived macrophages into osteoclasts both in vitro and in vivo, suggesting a RANKL-independent mechanism that is not inhibited by OPG [108,109].

In patients with RA, intracellular levels of IL-6 are typically high, and it is well-established that RA is an independent risk factor for osteoporosis [110]. Osteoporosis with severe alterations in cortical and trabecular bone microarchitecture, accompanied by a decrease in the number of osteoblasts and an increase in osteoclasts, is observed in IL-6 transgenic mice [111]. Conversely, in the IL-6^−/−^ mouse model, mice were protected from bone loss induced by a high-fat diet [112]. Anti-IL-6R antibodies have been shown to prevent collagen-induced destruction of bone structure and bone loss in arthritic mice [113]. Indeed, IL-6R antagonists have been found to reduce osteoclast production and decrease bone resorption in an arthritic mouse model. In addition to the direct action of IL-6, it indirectly induces the production of M-CSF and RANKL by FLS, T cells, or bone marrow mesenchymal stem cells, thereby promoting osteoclast-mediated bone resorption in RA [114,115].

The conclusions drawn from the in vivo study are highly intriguing. In a study investigating bone loss using transgenic mice with high IL-6 expression, it was observed that these mice had an increased number of osteoclasts in the tibial trabeculae and calvarial bones, as well as a decreased thickness of the bone cortex compared to WT mice [111]. Interestingly, it has also been noted that there is a trend towards an increase in the number of tartrate-resistant acid phosphatase (TRAP)-positive osteoclasts in IL-6^−/−^ mice compared to WT mice. However, it is worth mentioning that approximately 50% of IL-6^−/−^ mice (and approximately 8.5% of WT mice) exhibit osteoclast apoptosis. Consequently, IL-6^−/−^ mice ultimately display an increase in tibial trabecular bone mass [116]. These findings suggest that IL-6 is not the sole quantitative inducer of osteoclasts. This may be attributed to the fact that the presence of high levels of IL-6 supports osteoclasts to have an extended survival period and faster growth. Conversely, the absence of IL-6 deprives osteoclasts of this favorable survival environment, resulting in a significantly shorter lifespan for osteoclasts and ultimately leading to an increase in bone mass.

### 8.4. Effects of IL-17 on Osteoclasts

Aside from its role as a pro-inflammatory cytokine produced by T cells, IL-17 also acts as a stimulator of osteoclast production in vitro [117]. By inducing RANKL expression, IL-17 severely disrupts the RANKL/OPG balance, leading to increased production of osteoclasts and bone erosion in conditions such as arthritis and other bone-related diseases, ultimately resulting in skeletal lesions [51,118]. Additionally, IL-17 stimulates the production of RANKL by osteoblasts, which upregulates RANK levels and indirectly enhances the osteolytic activity of RANKL [51]. Furthermore, IL-17 directly blocks bone formation [119]. The results indicated that IL-17A exacerbates synovial inflammation and bone loss in collagen-induced inflammatory arthritis. Overexpression of IL-17 in the knee joint of type II collagen-immune mice (AdIL-17) significantly promotes the destructive capacity of osteoclasts and is accompanied by a significant anti-tartaric acid phosphatase activity at sites of bone erosion in the cortex, subchondral, and trabecular bone [117]. Clinical studies have provided evidence that antibodies targeting IL-17 can improve rheumatoid disease activity and reduce bone loss in patients [120,121,122]. Furthermore, elevated levels of IL-17 due to estrogen deficiency have been shown to correlate with low BMD [61]. IL-17 also serves as an important driver in the bone loss phenomenon caused by parathyroid hormone [123]. Interestingly, reports suggest that the role of IL-17 is not singular, as IL-17 produced by γδ T cells has been shown to have a role in promoting fracture repair [124,125].

Common cytokines contributing to osteoclast-mediated bone loss are shown in Table 1.

**Figure 3:**
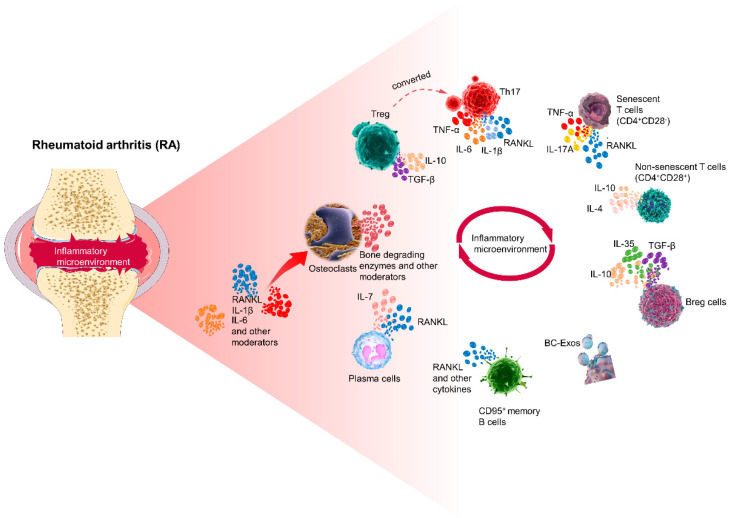
The interaction between T cells, B cells, and osteoclasts in RA. Within the joint microenvironment of RA, T cells, B cells, and interact in a complex and interconnected manner. Cytokines released by T cells and B cells, such as RANKL, IL-17, TNF-α, and IL-1β, promote osteoclast formation, while cytokines like IL-10 and TGF-β act to inhibit this process. Disruption of these delicate balances can lead to the establishment of a self-sustaining inflammatory environment, triggering abnormal activation of osteoclasts, excessive secretion of bone-degrading enzymes, and ultimately severe bone damage.

In summary, we have depicted the interplay among T cells, B cells, and osteoclasts in RA in Figure 3.

## 9. New Biomarkers for RA

According to the 2010 American College of Rheumatology (ACR)/European League Against Rheumatism (EULAR) classification criteria, RF and ACPA are widely recognized serological markers for diagnosing RA [135,136]. RF is found in approximately 50–80% of RA patients, with a specificity of around 66%. However, RF can also be present in other autoimmune conditions, systemic infections, and even in up to 10% of healthy individuals [135]. Despite this, there are still RA patients who test negative for both ACPA and RF. Therefore, in addition to traditional ACPA and RF tests, the utilization of new biomarkers for early detection of RA, risk assessment (prognosis), and treatment monitoring is essential. Below are some of the outlined biomarkers:

### 9.1. Anti-Malondialdehyde (MDA) and Anti-Malondialdehyde Acetaldehyde (MAA)

Anti-MDA and anti-MAA are a class of autoantibodies linked to oxidative stress and damage resulting from inflammation. In RA, where elevated levels of oxidative stress are common, these antibodies may play a crucial role by neutralizing pro-inflammatory factors and modulating the immune response. The presence of high oxidative stress leads to cellular and tissue damage, triggering an inflammatory cascade and joint degradation. Levels of anti-MDA and anti-MAA antibodies are associated with disease activity and joint damage severity in RA patients [135]. Furthermore, a positive correlation between anti-MAA antibodies and ACPA has been noted using multi-antigen arrays [137]. However, it is important to note that anti-MAA antibodies lack specificity, as they are also detected in patients with alcoholic hepatitis or cirrhosis and those with type 2 diabetes [138,139].

### 9.2. Anti-CII

Anti-CII antibodies recognize various epitopes in human RA and mouse models of arthritis, some of which are shared between species, including citrullinated-CII, C1, and U1 epitopes [139]. Higher titers of anti-CII antibodies have been observed in RA synovial fluid, along with more anti-CII antibody-producing B cells in RA synovium [140,141]. Moreover, studies have demonstrated that serum and synovial anti-CII IgG titers correlate with levels of acute-phase proteins and pro-inflammatory cytokines such as TNF-α and IL-6.

### 9.3. Anti-Mutated Citrullinated Vimentin (MCV)

MCV is a structural protein composed of microfilaments in the cytoskeleton and may undergo deamination in RA. In early RA, the sensitivity and specificity of anti-MCV (64% and 97%) are higher than those of RF [142]. A recent meta-analysis, encompassing 12 studies and 2003 RA patients, demonstrated a higher sensitivity of anti-MCV (68.6%) compared to anti-CCP (61.7%) in diagnosing RA, although anti-MCV’s specificity (94.2%) remained lower than that of anti-CCP (97.1%) [143]. Elevated levels of anti-MCV antibodies appear to indicate a rapid erosive disease in early RA and have been linked to active disease and poorer outcomes [144,145].

### 9.4. Other Biomarkers

Antibodies against the stress protein immunoglobulin heavy chain binding protein (BiP) (native and citrullinated) are present in 64–72% of patients with RA, with a specificity of 71% and a sensitivity of 73% [146,147]. Since BiP stimulates the proliferation of synovial T cells and polymorphonuclear cells, they have been implicated in the pathogenesis of RA [148,149]. These antibodies, a family distinct from ACPA, were first identified in 35–45% of RA patients, most of whom were ACPA-positive (49–74%) but also ACPA-negative (16–30%) [150,151]. In a recent study, anti-CarP antibodies had a sensitivity of 44% and a specificity of 89% for early arthritis, both lower than anti-CP and RF [152]. Therefore, anti-CarP antibodies can be used for early diagnosis, especially in ACPA-negative patients, as well as for identifying patients who require aggressive treatment [153,154]. How anti-CarP antibodies contribute to arthritis is unknown.

Hence, the quest for more specific biological markers holds significant clinical importance for early RA diagnosis, risk assessment, and treatment response monitoring. This pursuit also represents a crucial area of exploration for basic medical researchers.

## 10. Therapeutic Agents That Affect Osteoclasts or Alleviate Inflammation to Prevent Bone Erosion

### 10.1. Therapeutic Agents That Affect Osteoclasts

Several anti-bone resorption drugs have been identified to modify osteoclasts, playing a therapeutic role in the bone erosion and destruction that occurs during the course of RA, as outlined in part below.

#### 10.1.1. RANKL Monoclonal Antibody

RANKL serves as a crucial regulator in the differentiation of osteoclasts, which are produced by cells such as osteoblasts or immune cells. On the other hand, denosumab is a novel anti-bone resorption drug that acts as a humanized monoclonal antibody targeting RANKL. It was approved by the United States Food and Drug Administration (FDA) in 2010, making it the first and only targeted drug inhibiting the RANK ligand RANKL. Subsequently, on 23 November 2020, the Chinese National Medical Products Administration (NMPA) approved denosumab for marketing, specifically for the prevention of bone-related events in patients with bone metastases from solid tumors or multiple myeloma. Following subcutaneous administration of denosumab, bone resorption markers rapidly decrease to minimal levels within a few days. Moreover, bone formation markers can decrease by more than 60% over the subsequent months [155]. Administered twice yearly, denosumab enhances BMD and reduces the risk of vertebral, non-vertebral, and hip fractures in postmenopausal women with osteoporosis. Findings from the FREEDOM trial demonstrated that denosumab treatment led to a significant 68% reduction in vertebral fractures (RR 0.32, 95% CI 0.26–0.41) and a 40% reduction in hip fractures (HR 0.60, 95% CI 0.37–0.97) [156]. However, it is important to note a potential safety concern regarding denosumab. Clusters of vertebral fractures have been reported within 12 months after discontinuation of the drug. In the extended results of a phase Ⅲ study, it was found that 15% of women experienced vertebral fractures immediately after stopping denosumab, with two-thirds of these patients experiencing multiple fractures. This risk was particularly high in patients with pre-existing vertebral deformities and was directly proportional to the length of time denosumab was used prior to discontinuation [157].

According to a retrospective study, transitioning to another anti-resorptive therapy such as alendronate and zoledronic acid after discontinuing denosumab may have a preventive effect on bone loss. However, it should be noted that risedronate does not possess this function [158]. Additionally, the transition to teriparatide, a parathyroid hormone analog, is not recommended as it has been found to cause bone loss in the radius and hip [159]. Therefore, it is crucial to personalize a transition program prior to initiating denosumab in order to minimize the risk of fractures. Furthermore, emphasis should be placed on vitamin D supplementation prior to using denosumab to prevent hypocalcemia and potentially reduce the risk of fractures. The study also revealed that high-resolution quantitative computed tomography demonstrated partial bone repair (reduction in depth, width, and volume of erosion) after 6 months of denosumab treatment in patients with RA. Conversely, alendronate treatment was found to be ineffective in achieving the same results [160].

In a Japanese trial evaluating the inhibitory effect of denosumab on bone erosion in patients with RA treated with methotrexate, 350 patients with baseline methotrexate-treated RA were randomly assigned to receive denosumab at different intervals (every 6 months, every 3 months, or every 2 months) or placebo in a 1:1:1:1 ratio for 12 months. After 12 months, all denosumab doses showed significant suppression of radiological progression, as determined by the modified Sharp celiac score. The treatment group exhibited maintained BMD compared to the placebo group, and no effect on joint space narrowing was observed. Additionally, there were no increased adverse events reported [161]. Another study indicated that denosumab effectively reduced bone erosion even in patients receiving biological disease-modifying antirheumatic drugs (bDMARDs), corticosteroids, or bisphosphonates without major safety concerns. Radiographic analyses further confirmed the enhanced efficacy of combining denosumab with bDMARDs for the treatment of RA [162].

Romosozumab is a humanized monoclonal antibody that binds with and inhibits sclerostin through Wnt signaling, resulting in a dual effect of increasing bone formation while decreasing bone resorption [163]. In the findings of a randomized clinical pilot study, romosozumab treatment was demonstrated to be more effective than denosumab in enhancing lumbar spine BMD, potentially rendering it a viable option for patients who require a significant increase in lumbar spine BMD and are at a higher risk of fractures [164]. In patients with RA, no discernible difference in disease activity or joint damage was observed between romosozumab and denosumab treatments. However, there have been reports in the literature suggesting that romosozumab treatment may have adverse effects on disease activity and joint damage in RA patients. Nonetheless, it is worth noting that romosozumab treatment may not affect disease activity and joint damage in RA patients whose condition is relatively well-controlled [164].

#### 10.1.2. Bisphosphonates

The potent induction of apoptosis in osteoclasts by bisphosphonates has positioned them as a prominent treatment option for RA. The mechanism behind this apoptosis-inducing effect is associated with the presence of nitrogen in the bisphosphonate structure [165]. Initially, bisphosphonates bind to the bone matrix and are taken up by osteoclasts during the process of bone resorption. Nitrogen-free bisphosphonates undergo metabolism to form toxic ATP analogs, which subsequently trigger apoptosis in osteoclasts [165]. On the other hand, nitrogen-containing bisphosphonates inhibit the production of ATP analogs by suppressing the mevalonate pathway. Nevertheless, they are still capable of inducing apoptosis in osteoclasts in laboratory settings. While nitrogen-containing bisphosphonates impede the production of ATP analogs by inhibiting the mevalonate pathway, they have been observed to induce apoptosis in osteoclasts in laboratory settings. Studies have demonstrated the effectiveness of Zoledronate in reducing bone and cartilage destruction in both rat collagen-induced arthritis and TNF-α transgenic mouse models. However, when analyzing the number of osteoclasts in iliac crest biopsies from patients treated with bisphosphonates, no reduction in osteoclasts was observed, and the exact reason for this remains unclear [166,167]. Alternatively, bisphosphonates have been found to decrease systemic levels of tartrate-resistant acid phosphatase 5 (TRACP5) and Ctsk, suggesting that osteoclast apoptosis may occur systemically [168]. Moreover, it has been documented that the number of circulating osteoclast precursors decreases after more than one year of bisphosphonate treatment, and these reductions have been hypothesized to be associated with lower serum RANKL levels [169,170].

Our understanding of the effects of bisphosphonates on osteoclasts in vivo is currently limited, and further investigation is needed to better comprehend their mechanism of action beyond just inducing apoptosis in osteoclasts.

#### 10.1.3. JAK Inhibitor

Multiple studies have provided evidence linking the JAK/STAT signaling pathway to RA, suggesting a close relationship between the immune and skeletal systems. JAK inhibitors have demonstrated comparable efficacy in reducing joint inflammation when compared to biological disease-modifying antirheumatic drugs [171,172]. Although JAK inhibitors do not directly impact osteoclast precursors, they effectively inhibit osteoclast formation by suppressing the expression of RANKL on mesenchymal cells that support osteoclast function [173,174].

The in vitro studies have demonstrated that JAK inhibitors effectively inhibit the expression of pro-osteoclastogenic inflammatory factors, including IL-17, IFN-γ, IL-6, and TNF-α [175]. When human bone marrow mesenchymal stem cells were stimulated with RANKL and M-CSF and treated with JAK inhibitors, it resulted in reduced differentiation and activity of osteoclasts, as well as decreased expression of osteoclast markers (Ctsk and RANK) [176]. Furthermore, JAK inhibitors have been found to increase the expression of anabolic proteins such as Wnt1 and β-catenin in osteoblasts, promoting osteoblast formation and ultimately aiding in the restoration of bone mass to reverse bone erosion in RA [173]. Some JAK inhibitors have shown greater efficacy than TNF inhibitors in inhibiting bone erosion in RA patients, suggesting potential different mechanisms for preventing structural damage. In vivo experiments using a JAK inhibitor (ABT-317) demonstrated its ability to block the function of mature osteoclasts and impede the migration of osteoclast precursors to the bone surface, thus inhibiting LPS-induced bone resorption in mouse calvaria [177]. Preliminary studies in rats have also indicated that the administration of tofacitinib and baricitinib leads to increased bone mass, a decrease in the serum RANKL/OPG ratio, and enhanced osteoclast function [174]. A systematic review and meta-analysis involving over 4000 patients treated with tofacitinib for RA concluded that the treatment group exhibited significant improvement in signs and symptoms compared to other groups [178]. Additionally, the FDA has approved tofacitinib as a therapeutic option for RA, providing an alternative for patients who have not responded well to other medications [179].

#### 10.1.4. TNF-α Antagonist

Currently, there are five TNF-α antagonists approved for clinical use: adalimumab, a fully human monoclonal antibody (mAb); infliximab, a chimeric human/mouse mAb; etanercept, a soluble TNF-α receptor; certolizumab pegol; and golimumab. Numerous studies have demonstrated the ability of TNF-α antagonists to moderate or even prevent bone destruction, with most of these studies focusing on RA or ankylosing spondylitis. The mechanism of action behind this effect is beginning to be understood. TNF-α antagonists alleviate bone destruction by reducing the expression of RANKL and the number of osteoclast precursors in the bloodstream, thus restoring homeostasis with OPG [180]. Furthermore, TNF-α antagonists have been found to reduce bone damage in patients with erosive arthritis and psoriasis. A recent study observed that TNF-α inhibitors led to significant improvements in clinical symptoms and a rapid and substantial decrease in the number of osteoclast precursors in the peripheral blood of psoriasis patients with erosive arthritis [180].

#### 10.1.5. Selective Estrogen Receptor Modulators/Hormone Replacement Therapy

While estrogens are commonly used in the treatment of postmenopausal osteoporosis, it is important to note that they have been shown to directly inhibit the formation and function of osteoclasts at various stages, including osteoclastogenesis, resorption, and apoptosis [181]. Estrogen inhibits the activity of c-Jun, a protein involved in osteoclast precursor cell differentiation induced by RANKL, as well as basal c-Jun N-terminal kinase activity, thereby exerting a direct inhibitory effect on osteoclasts [182]. Additionally, estrogen has been found to downregulate αvβ3 integrins, which play a role in osteoclast differentiation in humans [181]. Moreover, mature osteoclasts have been shown to respond directly to estrogen [183,184]. Research has shown that tamoxifen directly inhibits the formation of osteoclasts, while raloxifene and ospemifene inhibit osteoclast activity by enhancing the expression of osteoblasts’ OPG [185,186]. Furthermore, estrogen has been found to downregulate lysosomal enzyme activity and production, which may explain the reduced uptake through the downregulation of proteins such as Ctsk and TRACP [187,188]. However, the inhibitory effect of estrogen on osteoclasts remains a topic of controversy, as some studies support its anti-osteoclastogenic effect while others reach the opposite conclusion. The specific mechanism underlying this discrepancy has yet to be elucidated.

#### 10.1.6. Ctsk Inhibitor

The only gene that has been identified as crucial for bone resorption in both mice and humans is Ctsk, a cysteine protease that is highly expressed in osteoclasts and plays a role in collagen breakdown [189]. It is worth noting that Ctsk also serves an important function in the immune system [190]. Odanacatib (ODN) is a specific inhibitor of Ctsk. The remarkable aspect of ODN is that it reduces the bone resorption capacity of osteoclasts to a certain extent while still preserving their activity and function. Furthermore, ODN has a milder impact on osteoblasts without disrupting baseline bone remodeling [30]. Research on ODN has also delved into its interaction with the immune system. For instance, the application of ODN has been shown to reduce the number of osteoclasts, macrophages, and T cells in periodontitis lesions, as well as the expression of toll-like receptors (TLRs). Additionally, ODN suppresses bacterial-induced immune responses and mitigates bone destruction in the affected area [191]. These findings suggest that inhibiting Ctsk may represent an effective therapeutic target for immune-related diseases accompanied by bone loss phenotypes, such as periodontitis, RA, and others.

In clinical trials, once-weekly application of ODN (oral, 50 mg/dose) consistently increased BMD in postmenopausal women with osteoporosis (n = 399) [192]. The LOFT (Long-Term Odanacatib Fracture Trial, NCT00529373), a phase Ⅲ follow-up study, was conducted to assess the long-term effects of ODN in postmenopausal women with osteoporosis. The results demonstrated that the baseline patient characteristics and changes in BMD over a 5-year period were comparable to the overall LOFT population in the subset analyzed (ODN n = 17, placebo n = 23 at baseline; ODN n = 112, placebo n = 104 at month 24; ODN n = 42, placebo n = 41 at month 36; ODN n = 27, placebo n = 20 at month 60). Qualitative assessment of biopsies showed no abnormalities. Notably, an increased number of osteoclasts were observed in the ODN group compared to the placebo group over time. In terms of bone remodeling, dynamic bone formation indices in trabeculae, intracortical, and intracortical surfaces were similar between ODN-treated and placebo-treated patients after 2 years of treatment [193]. Unfortunately, further development of ODN was discontinued due to its high risk of stroke.

### 10.2. Medications That Indirectly Suppress Osteoclast Activity by Reducing Inflammation

Given the strong connection between the immune system and osteoclasts, targeting osteoclast production by reducing inflammation is a promising therapeutic approach. In this regard, we will briefly outline two categories of drugs that effectively mitigate bone destruction in RA through this mechanism:

#### 10.2.1. Methotrexate (MTX)

As an “anchor drug” used alone or in combination therapy to treat RA, MTX is superior to other traditional synthetic DMARDs in alleviating arthritis symptoms, reducing disability, and delaying radiographic structural damage. MTX manages joint inflammation and alleviates symptoms by suppressing abnormal immune system activity and diminishing the release of inflammatory mediators. As a result of mitigating the inflammatory response, bone destruction is indirectly diminished.

The immune system in RA is characterized by abnormal expression of Th cells among CD4^+^ T cells, including Th1, Th2, and Th17 cells, and Treg subpopulations [194]. Many studies have demonstrated that various CD4^+^ subpopulations in RA tend to be pro-inflammatory. Within the peripheral blood of RA patients, there is an increased prevalence of Th1, Th2, and pro-inflammatory Th17 cells, while the prevalence of Tregs is significantly decreased [194,195]. The pathophysiological mechanism of RA may be linked to Th1 cell activation and the lack of Th2 cell differentiation, resulting in inflammation [196]. The overall effect of a shift towards a pro-inflammatory Th17/Treg ratio is T-lymphocyte activation. Th17 cells are notably proinflammatory and participate in various inflammatory conditions by regulating the production of IL-17, TNF-α, and IL-6. Their prevalence may contribute to inflammation, cartilage destruction, and concurrent bone erosion in RA [195]. MTX may aid in restoring immune balance by reducing arthritis through decreased Th1 cell production and improved Th2 cytokine levels [197]. Studies have revealed that MTX treatment facilitates the normalization of Treg cell suppressor function in RA patients, linked with increased Foxp3 and CTLA-4 protein expression. This is achieved through a mechanism wherein MTX reduces DNA methyltransferase-1 (DNMT-1) gene expression and methylation of the upstream enhancer of Foxp3 [198]. The expression of CD4^+^CD25^+^Foxp3^+^ was significantly higher in RA patients treated with MTX compared to untreated patients. Additionally, as a transcription factor, the upregulation of Foxp3 promotes the secretion of immunosuppressive factors such as IL-10, contributing to negative immunomodulation and the maintenance of autoimmune tolerance. Following MTX treatment, the circulating Th17/Treg ratio in RA patients exhibited a significant decrease. Consequently, MTX may enhance the management of RA by influencing the normalization of cell distribution, including Th17, Treg, and their related cytokines [194].

#### 10.2.2. Biologic Response Modifiers

Biologic response modifiers, such as anti-TNF-α drugs (e.g., infliximab) and IL-6 inhibitors (e.g., tocilizumab), target specific inflammatory mediators and modulate immune system function to reduce joint inflammation and slow disease progression, in addition to the direct impact on osteoclasts mentioned earlier for anti-TNF-α drugs. By attenuating the inflammatory response, these drugs can also indirectly reduce bone resorption. In the treatment of RA, biologic agents not only help reduce cartilage damage but also alleviate local and systemic bone loss. Many studies on RA have reported positive effects on BMD following treatment with biologic agents. While most studies have focused on bone markers, some have also assessed BMD and fracture risk [199]. An exploratory analysis of a 12-month randomized, double-blind, placebo-controlled study indicates a causal relationship between inflammation and bone loss in RA. The anti-inflammatory effect of infliximab was sufficient to halt inflammatory bone loss at the hip but not in the spine and hand [200]. Additionally, there have been reports indicating that tocilizumab inhibits BMD in patients with active RA who are receiving MTX treatment, and it also increases BMD in patients with baseline osteopenia [201].

We have summarized some medications in Table 2, details can be found in the table below:

## 11. Conclusions

T cells, B cells, and osteoclasts collectively play crucial roles in the exacerbation of inflammation, bone erosion, and cartilage destruction in the progression of RA disease. Current therapeutic strategies predominantly focus on mitigating inflammation and stemming joint damage. Thus, gaining further insight into the interactions and characterization of immune cells and osteoclasts in RA pathology is vital to discovering new therapeutic strategies. T cells and B cells, as the primary leaders of cellular and humoral immunity respectively, also play a significant role in maintaining inflammation in RA. Activated T cells, upon exposure to antigens presented by antigen-presenting cells, assist in activating B cells to produce ACPAs and RF. Moreover, the immune-activated joint microenvironment disrupts the balance between Th17 and Treg subpopulations, with Th17 cells believed to play a key role in joint inflammation and bone resorption in osteoclasts. Conversely, Treg cells alleviate inflammation and inhibit osteoclasts from functioning. Interestingly, the Th17/Treg cell balance axis may serve as a promising therapeutic target for reducing RA inflammation and bone erosion due to the potential interchange in roles based on the inflammatory environment. Studies have shown that senescent T cells promote osteoclasts, leading to further risk of bone erosion. Different B cell types, including plasma cells and memory B cells, also contribute to the inflammatory response and hyperactivity of osteoclasts. Additionally, IL-6, IL-1β, and TNF-α secreted by T and B cells stimulate osteoclasts. IL-6 and TNF-α can induce osteoclast differentiation in the absence of RANKL, and anti-TNF-α has a better therapeutic effect in alleviating RA. RANKL, an important osteoclast stimulator, not only regulates the roles and functions of T cells and B cells but also highlights the intersection between T cells, B cells, and osteoclasts, underscoring the inseparability of inflammatory immunity and bone erosion in RA. Given that not all RA patients respond well to existing therapeutic strategies, targeting the immune system to increase beneficial T/B cell subsets, suppress destructive T/B cell subsets, or inhibit the function of osteoclasts without compromising the osteoclasts/osteoblasts axis can be considered as part of the therapeutic strategy, with concomitant targeted therapy making sense.

In conditions like RA, novel biomarkers can aid in early detection, evaluating patient risk and prognosis, and monitoring response to treatment. Certain biomarkers may even correlate with disease activity levels, joint damage severity, and the rate of disease progression, thereby offering clinicians supplementary diagnostic and treatment choices. As technology continues to progress and clinical studies become more intensive, we anticipate a greater utilization of new biomarkers in clinical practice, paving the way for improved disease management and personalized treatment.

## Figures and Tables

**Table 1:** Effect of related cytokines on osteoclasts.

Cytokines	Sources	Effect on Osteoclast Differentiation	Function in Bone Homeostasis	Ref.
IL-4	Th2 cells	Suppression	Reduce the expression of TRAP and RANKL to inhibit bone resorption.	[126]
IL-6	Th2 cells	Promotion	Boost the expression of M-CSF and RANKL, promoting bone resorption.	[115]
IL-7	Thymic stromal cells	Promotion	Promote RANKL-mediated osteoclast bone resorption.	[101,127]
IL-10	Treg cells Breg cells	Suppression	Downregulate NFATc1, TNF-α, and IL-6 production; induce OPG expression.	[128,129]
IL-17	Th17 cells	Promotion	Promote RANKL expression levels, trigger a cascade of pro-inflammatory responses, and amplify the effects on osteoclasts.	[59,130]
IL-35	Treg cells, B cell	Suppression	Reduce IL-17 levels and indirectly inhibit RANKL expression.	[131,132]
TNF-α	Th17 cells, Macrophages	Promotion	Indirectly stimulate osteoclast production through RANKL production by B cells; enhance RANK/RANKL expression and reduce OPG production to induce osteoclast differentiation.	[90,133]
IFN-γ	Th1 cells	Suppression	Inhibit RANKL- and TNF-α-induced osteoclast differentiation; stimulate osteoclast apoptosis.	[54,134]
RANKL	Th cells	Promotion	Directly activate osteoclast function-related genes through binding to RANKL.	[133]

**Table 2:** Drugs affecting osteoclasts to reduce bone erosion.

Classification	Drug(s)	Modes of Action and Characteristics	Ref.
RANKL monoclonal antibody	Denosumab	Blocks RANKL activation on the surface of osteoclasts and their precursors and inhibits osteoclast activation and maturation.	[156,158]
	Romosozumab	Binds with and inhibits sclerostin through Wnt signaling, which has a dual effect on increasing bone formation and decreasing bone resorption.	[164]
Bisphosphonate	Zoledronate	Induces osteoclast apoptosis and reduces systemic RANKL levels.	[167,168]
JAK inhibitor	Baricitinib	Reduces RANKL and IL-6 levels; does not affect osteoclast function and activity; decreases RANKL levels produced by T lymphocytes.	[174]
JAK inhibitor	Tofacitinib	Reduces the ratio of RANKL/OPG in serum.	
JAK inhibitor	CYT387	Attenuates the formation of osteoclasts; suppresses the bone reabsorption function and the expression and activation of osteoclasts; inhibits the intracellular Ca^2+^ influx.	[202]
TNF-α antagonist	Infliximab	Reduces RANKL expression and osteoclast precursors.	[203]
Selective estrogen receptor modulator	Tamoxifen	Inhibits osteoclast occurrence and bone resorption; promotes osteoclast apoptosis.	[166,187]
Ctsk inhibitor	Odanacatib	Reduces the bone resorption capacity of osteoclasts but maintains the activities and functions of osteoclasts with less impact on osteoblasts; does not disrupt bone remodeling at the baseline level.	[30]
Traditional synthetic DMARD	MTX	Decreases the production of Th1 cells, improves the levels of Th2 cytokines, and reduces the Th17/Treg ratio; osteoclasts are indirectly inhibited.	[197,198]
Biologic response modifiers	Infliximab, tocilizumab	Target specific inflammatory mediators to reduce inflammation and indirectly inhibit osteoclasts.	[199]
